# Anti-ganglioside antibodies and celiac disease

**DOI:** 10.1186/s13223-021-00557-y

**Published:** 2021-05-28

**Authors:** Alessandro Granito, Francesco Tovoli, Alberto Raiteri, Umberto Volta

**Affiliations:** 1 Department of Medical and Surgical Sciences, University of Bologna, Bologna, Italy; 2 Division of Internal Medicine, Hepatobiliary and Immunoallergic Diseases, IRCCS Azienda Ospedaliero-Universitaria Di Bologna, Bologna, Italy

Dear Editor,

We read with great interest the article by Cutillo et al. analyzing the multiple roles of gangliosides and their key components, sialic acids, in the protection of human and microbial cells from host immune response, and their potential to serve as targets for autoimmunity [1].

In their discussion of anti-ganglioside antibodies and analysis of the various human pathologies in which anti-ganglioside antibodies have been reported, the authors mention celiac disease (CD) as a condition associated with the presence of anti-GM1 antibodies. They also state that the triggering factor that induces anti-ganglioside antibodies generation is unknown. However, the authors support the hypothesis that the formation of complexes between gliadin and GM1 ganglioside leads to the generation of antibodies to GM1 as a “secondary product”. In this respect, CD can be considered an autoimmune disease where, unusually, several pathogenetic factors are well known, i.e., the extrinsic trigger (gliadin), a close genetic background (HLA-DQ2 or -DQ8), and a highly specific immune response directed to a well-characterized autoantigen (tissue transglutaminase). Our data on the presence of anti-neuronal antibodies to central/enteric nervous systems provide further support for the autoimmune hypothesis of neurological dysfunction in CD patients [2–4].

We have previously described in 2006 our own experience on the prevalence of a wider range of anti-ganglioside antibodies and their clinical significance in CD patients [5, 6].

Using a commercially available ELISA kit (IMMCO Diagnostics, Buffalo, NY, USA), we studied anti-GM1, anti-GD1b, and anti-GQ1b serum IgG and IgM antibodies in 22 adult patients (median age 35, range: 19–56 years; three males, 19 females) with CD and neurological manifestations, including eight cases of idiopathic cerebellar ataxia, seven cases with epilepsy (without cerebral calcifications), two with multiple sclerosis, three with attention/memory impairment, and two with peripheral neuropathies.

In all cases, diagnosis of CD was confirmed by endoscopic duodenal biopsy, revealing different grades of villous atrophy (from 3a to 3c, according to the modified Marsh classification). In all CD patients, intestinal villous atrophy was associated with a positivity for serological CD markers (anti-endomysial and/or anti-tissue transglutaminase antibodies) further supporting the diagnosis of CD. All available data, regarding CD diagnosis, diagnostic work-up, histopathology and treatment were obtained from the hospital digital database.

In addition, anti-ganglioside antibodies status was assessed in 30 patients with CD without neurological dysfunction (median age 37 years, range 17–59 years, eight males, 22 females), 20 patients with neurological disorders (seven with idiopathic cerebellar ataxia, seven with epilepsy, four with peripheral neuropathy, one with paraneoplastic syndrome and subacute cerebellar atrophy, and one with amyotrophic lateral sclerosis), 50 patients with immune system disorders (six with Crohn’s disease, four with ulcerative colitis, 10 with autoimmune hepatitis, 20 with primary biliary cholangitis, and 10 with the calcifications, Raynaud’s phenomenon, esophageal hypomotility, sclerodactyly, and telangiectasia (CREST) syndrome, and 20 blood donors with comparable age and sex demographics. The study was approved by the local Ethics Committee and all patients and controls gave their informed consent before.

Our anti-ganglioside antibodies assessment results are summarized in Fig. 1. At least one of the three anti-ganglioside IgG antibodies tested for (anti-GM1, anti-GD1b, anti-GQ1b) was found in 64% of CD patients with neurological dysfunction compared to 30% of CD patients without neurological symptoms, 50% of neurological patients without CD, 20% of autoimmune controls and none of the healthy controls (p = 0.02, p = ns, p = 0.003 and p = 0.0001, respectively).

**Fig. 1:**
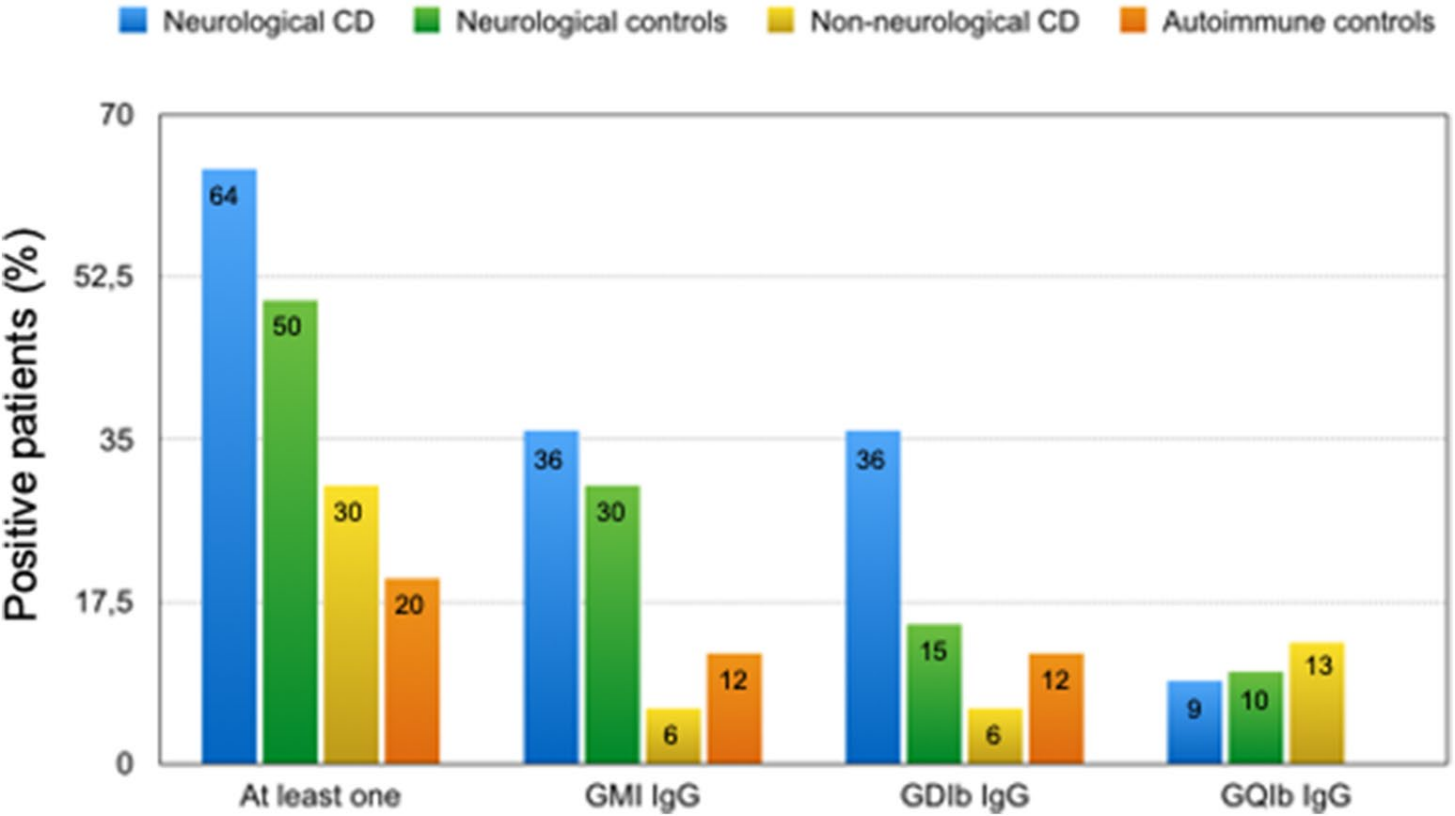
Immunoglobulin G (IgG) antibodies to GM1, GD1b, and GQ1b, expressed as the percentage of patients in each study population that was positive for at least one IgG antibody: CD with a neurological disorder vs CD without neurological disorder, control group with a neurological disorder, and control group with an autoimmune disorder: p = 0.02, p = ns, and p = 0.003, respectively. GM1 IgG: CD with a neurological disorder vs CD without neurological disorder, control group with a neurological disorder, and control group with an autoimmune disorder: p = 0.01, p = ns, p = 0.02 respectively. GD1b IgG: CD with a neurological disorder vs CD without neurological disorder, control group with a neurological disorder, and control group with an autoimmune disorder: p = 0.01, p = ns, p = 0.02, respectively. GQ1b IgG: No significant difference was found; Fisher’s exact test

Analysis of individual reactive antibody types showed that both anti-GM1 and anti-GD1b IgG were significantly more frequent in CD patients with neurological dysfunction than in CD patients without neurological symptoms, autoimmune controls, and blood donors. No significant difference between groups was found for anti-GQ1b IgG.

Among the neurological patients with CD, six of the seven with epilepsy, two of the three with attention deficit/memory impairment syndrome, three of the eight with idiopathic cerebellar ataxia, one of the two with multiple sclerosis, and both patients with peripheral neuropathy had anti-ganglioside IgG antibodies.

Of these 14 patients, 11 showed reactivity against only one ganglioside, two showed reactivity to two gangliosides, and one patient showed reactivity to all three gangliosides. Within the group with neurological disorders but without CD, four of the seven with idiopathic cerebellar ataxia, four of the seven with epilepsy, and two of the four with peripheral neuropathy were positive for IgG antibodies to gangliosides.

In patients with autoimmune diseases, anti-ganglioside antibodies were found in three of the six with Crohn’s disease, one of the four with UC, two of the 10 with AIH, two of the 20 with PBC, and two of the 10 with CREST syndrome.

Ganglioside reactivity, expressed in terms of Enzymatic Units (AEU) associated with anti-GM1 and anti-GD1b IgG were significantly higher in CD patients with neurological disorders (anti-GM1: median 20.35 AEU, range 2.6–136.5 AU; anti-GD1b: median 16.5 AEU, range 5.9–79.4 AEU) than in CD patients without neurological disorders (anti-GM1: median 16.2 AEU, range 5.9–35.5 AEU; anti-GD1b: median 12.05 AEU, range 5.9–33.1 AEU) (p = 0.04 and p = 0.02, respectively), autoimmune control patients (anti-GM1: median 13.1 AEU, range 5.3–41.2 AEU; anti-GD1b: median 11.1 AEU, range 5.9–33.1 AEU) (p = 0.007 and p = 0.02, respectively) and healthy blood donors (anti-GM1: median 12.5 AEU, range 5.0–24.0; anti-GD1b: median 9.4 AEU, range 1.1–18.0 AEU) (p = 0.009 and p = 0.0001, respectively).

No correlation was found between AEU of antibodies to gangliosides and the severity of villous atrophy. Of note, 8 (47%) of the 17 patients with CD and neurological manifestations who were positive for at least one anti-ganglioside IgG antibody became negative for the antibody after 1 year of strict adherence to a gluten-free diet.

Interestingly, anti-ganglioside IgM antibodies, although at a lower prevalence than anti-ganglioside IgG antibodies and without any significant difference among the various groups studied, were confined to three cases of epilepsy within the CD group with neurological dysfunction.

CD patients without neurological manifestations, as expected, during follow-up under gluten free diet tested negative for autoantibody markers of CD and exhibited no other autoantibodies.

The first description of anti-ganglioside antibodies in CD patients dates back to 2002 by Alaidini et al. who reported a positivity for at least one autoantibody directed against GM1, GM2, GD1a, GD1b, GT1b, and GQ1b gangliosides in 6 CD patients with peripheral neuropathy, thus assuming that neuropathy of CD may be autoimmune and associated with anti-ganglioside antibodies [7].

After our 2006 study, other authors confirmed the presence of anti-ganglioside antibodies in celiac disease and their potential pathogenetic role in other autoimmune and non-autoimmune neurological disorders (Table 1).

**Table 1:** Prevalence of antiganglioside antibodies in celiac disease and other autoimmune and non-autoimmune diseases

Study (reference)	Disease	Anti-ganglioside antibodies tested	Prevalence (%)/clinical significance
Cats EA et al. 2010 (Neurology; 75:1961–1967)	MMN	IgG, and IgA antibodies to GM1, GM2, GD1a, GD1b, GM1b, GT1a, GT1b, GQ1b, GalNAc-GD1a	Anti-GM1^^^: IgM: 43%, IgG: 1%, IgA: 5%
Lucchetta M et al. 2010 (Muscle Nerve; 41:50–53)	Type 1 Diabetes	IgG or IgM antibodies to GM1, GM2, GM3, GD1b, GD1a, GD3	24% (one or more gangliosides)
Mostafa GA et al. 2011 (J Neuroinflammation; 8:39)	Autistic children	Anti-ganglioside M1	74%*
Labrador-Horrillo M et al. 2012 (Lupus.;21:611–615)	SLE with neuropsychiatric manifestations	GM1, GM2, GM3, asialo-GM1 GD1a, GD1b, GD3, GT1b, GQ1b	15% IgM asialo-GM1, 6% positive for other anti-ganglioside antibodies (GM1, GM2, GM3, GD1b, GT1b, GD3, mainly IgM)
Kim JK et al. 2014 (J Clin Neurol; 10:94–100)	GBS	IgG and IgM antibodies to gangliosides GM1, GM2, GM3, GD1a, GD1b, GD3, GT1a, GT1b, and GQ1b	50% positive for IgG or IgM antibodies against various gangliosides
Kollewe K et al. 2015 (PLoSOne; 10:e0125339)	Amyotrophic Lateral Sclerosis	IgG and IgM antibodies to asialo1 (GA1), GM1, GM2, GD1a, GD1b, GQ1b	IgG: 10.7% IgM: 17.9%
Ge S et al. 2016 (Diabetes Res Clin Pract; 115:68–75)	Diabetic peripheral neuropathy	Anti-GS IgM anti-GS IgG	anti-GS Ab levels positively correlated with DPN
Przybylska-Feluś M et al. 2016 (Pol Arch Med Wewn; 126:763–771)	Celiac disease	Anti-GM1	Significantly higher median level of anti-GM1 antibodies than controls^§^
Saccomanno D et al. 2017 (Scand J Gastroenterol; 52:409–413)	Celiac disease	IgM, IgG, and IgA to GM1, GM2, GM3, GD1a, GD1b, GD3, GT1a, GT1b, GQ1b, sulfatide	Anti-sulfatide IgG antibodies (36%)

*Anti-GS IgM Ab and anti-GS IgG Ab*: anti-ganglioside IgM and IgG antibodies, *DPN* diabetic peripheral neuropathy, *GBS* Guillain-Barré syndrome, *SLE* systemic lupus erythematosus, *MMN* multifocal motor neuropathy

^*^Serum levels of anti-ganglioside M1 antibodies were significantly higher in autistic children with severe autism (63%) than those with mild to moderate autism (37%), p = 0.001. Moreover, serum anti-ganglioside M1 antibodies had significant positive correlations with Childhood Autism Rating Scale

^^^Patients with MMN with anti-GM1 IgM antibodies had more severe weakness (p < 0.01), more disability (p < 0.01), and more axon loss (p = 0.05) than patients without anti-GM1 IgM antibodies. Anti-GM1 IgM antibody titers correlated with Medical Research Council scores (correlation coefficient = 0.43; p < 0.0001). Anti-GD1b IgM antibody activity was associated with reduced vibration sense (p < 0.01)

^§^1.38 ng/ml [0.98–2.03 ng/ml] vs 0.81 ng/ml [0.35–1.15 ng/ml], (p < 0.001, the Mann–Whitney test)

Interestingly, a molecular mimicry between some microbial antigens, such as lipo-oligosaccharides of *Campylobacter jejuni* and the gangliosides has been hypothesized as a possible mechanism by which anti-ganglioside antibodies are generated, thus reflecting an abnormal immune response to microbiota antigens [8, 9].

Our results, which detected anti-ganglioside antibodies beyond anti-GM1, confirm and expand upon previously identified antineuronal antibodies (e.g., Hu-like and Yo-like detected by indirect immunofluorescence) in patients with CD and neurological complications, confirming the hypothesis that anti-ganglioside antibodies may result from an immunological disorder underlying CD [2, 4, 10].

In conclusion, our data support data described by Cutillo et al. on a potential pathogenic role of anti-ganglioside antibodies in immuno-mediated neurological disorders and provide evidence that detection of anti-ganglioside antibodies could indicate associated neurological symptoms in CD patients. Anti-ganglioside antibodies may therefore represent immunological markers for neurological dysfunction in CD patients and should be included in the work-up of CD patients.

## Data Availability

Not applicable.

## Abbreviations

CD: Celiac disease
AEU: Antiganglioside enzymatic units
CREST: The calcifications, Raynaud’s phenomenon, esophageal hypomotility, sclerodactyly, and telangiectasia syndrome
